# CycA-Dependent Glycine Assimilation Is Connected to Novobiocin Susceptibility in Escherichia coli

**DOI:** 10.1128/spectrum.02501-22

**Published:** 2022-11-15

**Authors:** Hongmei Shi, Ling Zhang, Jing Gu, Jinyue Li, Zixiang Liu, Jiao-Yu Deng

**Affiliations:** a Institute of Agro-Products Processing and Nuclear Agricultural Technology, Hubei Academy of Agricultural Sciences, Wuhan, China; b CAS Key Laboratory of Special Pathogens and Biosafety, Wuhan Institute of Virology, Chinese Academy of Sciences, Wuhan, China; c University of Chinese Academy of Sciences, Beijing, China; d Guangdong Province Key Laboratory of TB Systems Biology and Translational Medicine, Foshan, China; University of Minnesota

**Keywords:** serine hydroxymethyltransferase, threonylcarbamoyl-AMP synthase, novobiocin susceptibility, glycine metabolism, *Escherichia coli*

## Abstract

Escherichia coli serine hydroxymethyltransferase (GlyA) converts serine to glycine, and *glyA* mutants are auxotrophic for glycine. CycA is a transporter that mediates glycine uptake. Deleting *glyA* in E. coli strain W3110 led to activation of CysB, which was related to novobiocin (NOV) susceptibility. Moreover, deleting *glyA* resulted in increased sensitivity to NOV, and this could be reversed by high concentrations of glycine. Reverse mutants of Δ*glyA* were selected and one of them had a mutation in *yrdC*, the gene encoding threonylcarbamoyl-AMP synthase. Subsequent proteome analysis showed that deleting *glyA* led to increased expression of TcyP and TdcB, making this bacterium dependent on CycA for glycine assimilation. Furthermore, deleting *cycA* in a Δ*glyA* background caused a severe growth defect on Luria-Bertani medium, which could be complemented by high concentrations of exogenous glycine. Mutation of *yrdC* led to decreased expression of TdcB but increased expression of ThrA/B/C and LtaE, which favored the conversion of threonine to glycine and thus avoided the dependence on CycA. Correspondingly, deleting of *tcyP*, *tdcB*, or *gshA* could reverse the NOV-sensitive phenotype of Δ*glyA* mutants. Overexpression of *cycA* resulted in increased sensitivity to NOV, whereas deleting this gene caused NOV resistance. Moreover, overexpression of *cycA* led to increased accumulation of NOV upon drug treatment. Therefore, inactivation of *glyA* in E. coli led to CycA-dependent glycine assimilation, which enhanced the accumulation of NOV and then made the bacterium more sensitive to this drug. These findings broaden our understanding of glycine metabolism and mechanisms of NOV susceptibility.

**IMPORTANCE** Novobiocin (NOV) has been used in clinical practice as an ATPase inhibitor for decades. However, because it has been withdrawn from the market, pharmaceutical companies are searching for other ATPase inhibitors. Thus, probing the mechanisms of susceptibility to NOV will be beneficial to those efforts. In this study, we showed that inactivation of *glyA* in E. coli led to CycA-dependent glycine assimilation, which accompanied the accumulation of NOV and thereby increased the sensitivity to this drug. To date, this is the first report demonstrating the linkage between glycine assimilation and NOV susceptibility, and it is also the first report showing that YrdC is able to modulate the metabolic flux of threonine.

## INTRODUCTION

As a member of the coumarin family of antimicrobial drugs, novobiocin (NOV) inhibits the ATPase activity of bacterial DNA gyrase and topoisomerase IV (1), which are both validated drug targets. Although NOV has been withdrawn from the marketplace since 2011, pharmaceutical companies are still developing other ATPase inhibitors (2, 3). Low permeability across the outer membrane has been considered the major reason why NOV was ineffective against Gram-negative bacteria. However, recent studies have shown that some peptides can be used to repurpose NOV as an effective antibacterial agent against Gram-negative bacteria (4, 5).

Glycine is an essential amino acid required for synthesizing proteins, purines, glutathione, and other amino acids (6–9). Intracellular glycine can be synthesized by serine hydroxymethyltransferase (GlyA) or l-threonine aldolase (LtaE), transported by the glycine transporter CycA or glycine-containing substance transporters from the external environment (10–17). When glycine is present in a cell, the glycine cleavage system (GCV enzyme complex) hydrolyzes it (18–21). GlyA converts serine to glycine, transfers a methyl group to tetrahydrofolate, and thus forms 5,10-methylene-tetrahydrofolate (5,10-mTHF). *glyA* is essential for growth on glycerol minimal medium, and *glyA* mutants are auxotrophic for glycine (10, 22). The low-specificity l-threonine aldolase (LtaE) can act on l-threonine and l-allo-threonine, as well as l-threo-phenylserine and l-erythro-phenylserine, to form glycine (23). LtaE may serve in an alternative pathway for glycine biosynthesis when the major pathway is disrupted by mutation in *glyA*, but an *ltaE* mutant has been shown to have no growth defect when grown on glucose as the sole source of carbon (23).

CycA is an inner membrane protein which mediates the uptake of d-serine, d-alanine, and glycine. This protein also contributes to l-alanine uptake, is the major transporter for β-alanine uptake, and has been shown to be implicated in the uptake of d-cycloserine (15, 17, 24–26). *cycA* mutants are defective in the uptake of d-serine, d-alanine, and glycine (17, 26). CycA-mediated transport of glycine has also been implicated in the regulation of the glycine cleavage system (27). The GCV enzyme complex is important for balancing a cell’s requirements for glycine used in protein and purine biosynthesis and for C_1_ units used in various biosynthetic and methylation reactions (19, 21, 28).

In this study, we found that deleting *glyA* in Escherichia coli strain W3110 led to increased susceptibility to NOV, which could be complemented by introducing an intact copy of *glyA*. Although lower concentrations of glycine (10 μg mL^−1^) could support the growth of E. coli W3110 Δ*glyA* mutants on minimal medium, only higher concentrations of glycine (≥75 μg mL^−1^) could fully complement this NOV-sensitive phenotype, indicating a linkage of glycine assimilation and NOV susceptibility in E. coli. Subsequently, microbiological and biochemical methods, in combination with genomic, transcriptomic, and proteomic approaches, were used to test this hypothesis.

## RESULTS

### Deletion of *glyA* in E. coli W3110 resulted in activation of *CysB*.

**FIG 1:**
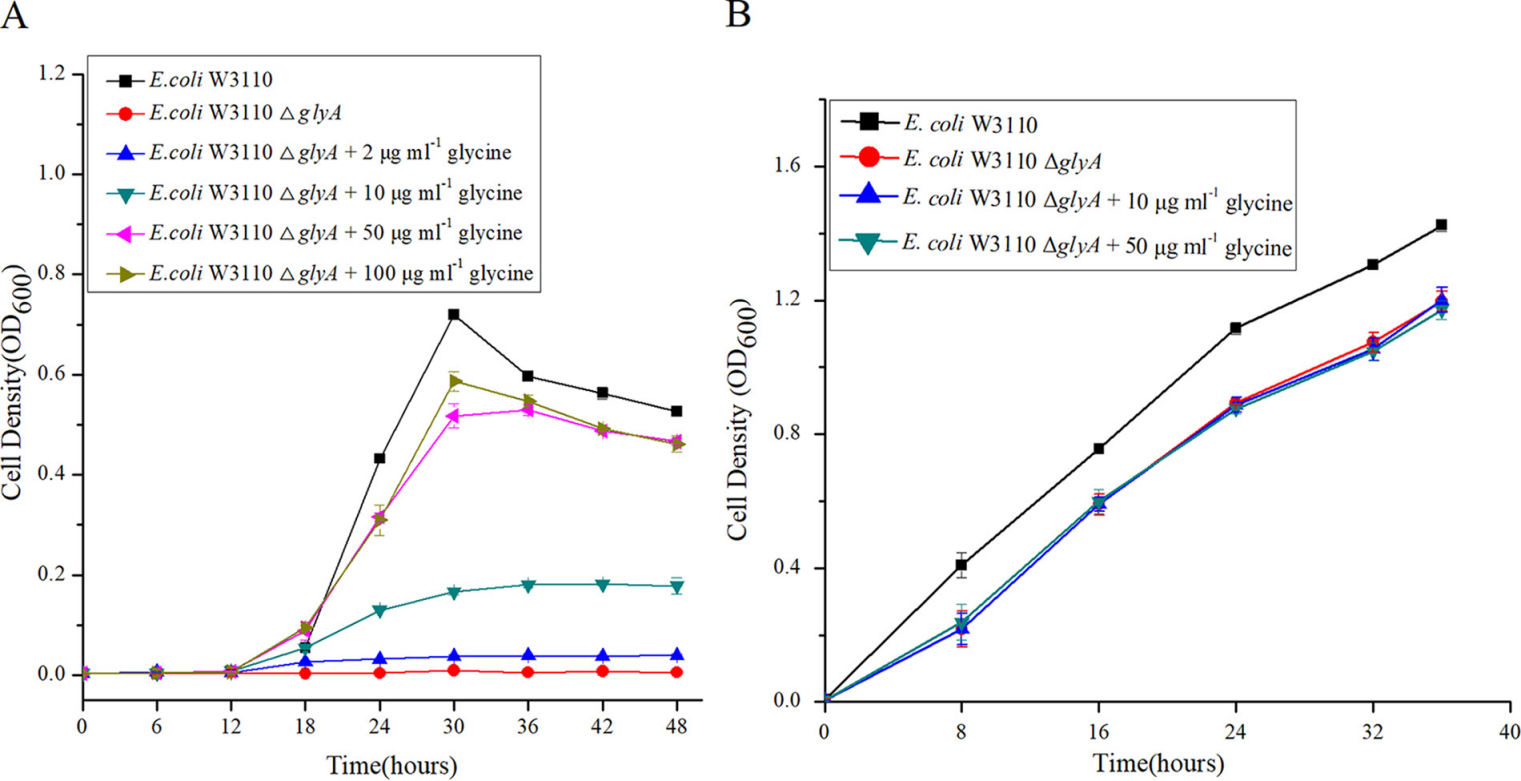
Growth of E. coli W3110 and E. coli W3110 Δ*glyA* on E medium (A) and on LB medium (B). The initial concentration of cells was 10^5^ CFU mL^−1^. Stationary cultivation was applied at 37°C, and the light absorbance of cultures was measured at 600 nm every 6 or 8 h. Data presented are means ± standard deviations (SD) from three independent experiments.

**FIG 2:**
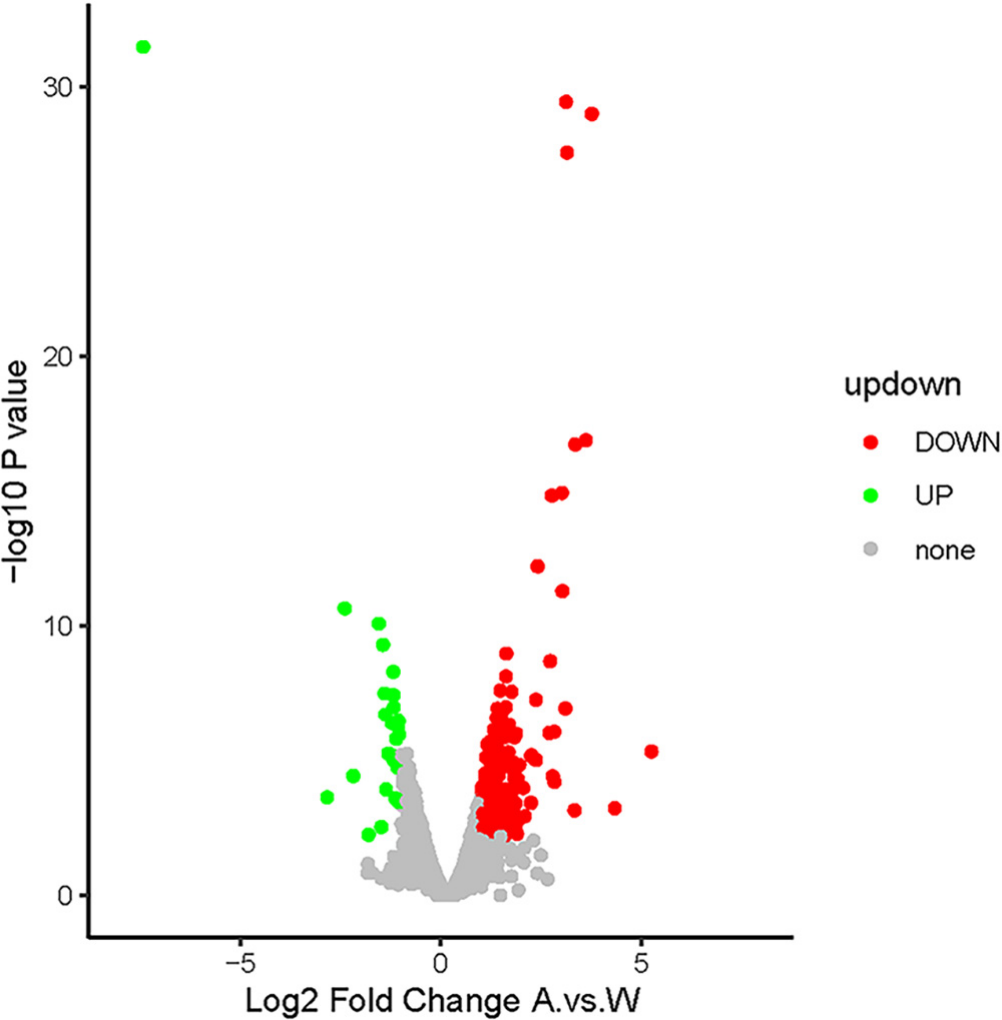
Volcano diagram of differentially expressed genes between E. coli W3110 and E. coli W3110 Δ*glyA*. The abscissa represents the difference multiple (log_2_ value) of the differential protein; the vertical axis represents the *P* value (−log_10_ value); the black represents the protein with insignificant difference, the red represents the downregulated protein, and the green represents the upregulated protein. Differentially expressed genes were selected only if the false-discovery rate *q* value was ≤0.05 and fold change was ≥2.

**FIG 3:**
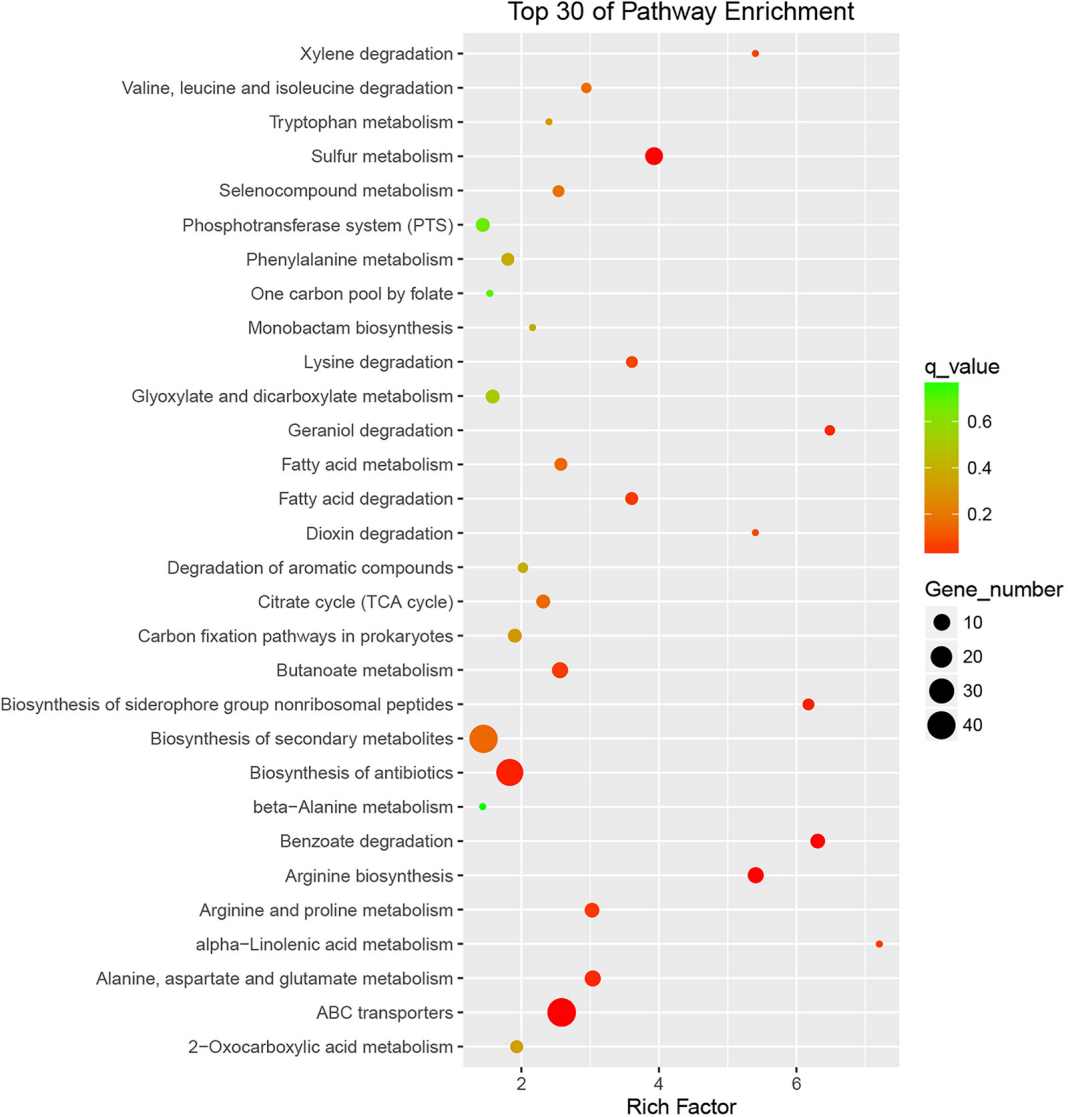
KEGG enrichment scatterplot of differentially expressed genes between E. coli W3110 and E. coli W3110 Δ*glyA*. Rich factor was calculated as follows: (number of differential genes in GO term)/(number of genes contained in GO term). The larger the rich factor, the greater the enrichment. The *q* value was the *P* value corrected for multiple-hypothesis testing. The smaller the value, the more significant the enrichment. Only the top 30 GO entries of the rich factor are shown.

Consistent with a previous report (22), deletion of *glyA* in E. coli W3110 resulted in a glycine auxotrophic phenotype on E minimal medium, which could be complemented using exogenous glycine (≥50 μg mL^−1^) (Fig. 1A). This Δ*glyA* strain also showed a slight growth defect on Luria-Bertani (LB) medium, which could not be complemented by supplementing the medium with 50 μg mL^−1^ glycine (Fig. 1B). To probe the adaptation of this bacterium in the absence of *glyA*, comparative transcriptomic analysis was performed. Three biological replicates were used for RNA sequencing (RNA-seq), and *P* and *q* values were calculated. Differentially expressed genes were selected only if the false-discovery rate *q* value was ≤0.05 and the fold change was ≥2 (Fig. 2). A principal-component analysis (PCA) map of the RNA-seq results is provided in Fig. S1 in the supplemental material. Differentially expressed genes of the top 30 Gene Ontology (GO) entries for rich factor are shown in the KEGG enrichment scatterplot (Fig. 3). The results showed that the expression levels of many genes involved in sulfur metabolism increased significantly (Table 1), implying the activation of CysB, the major regulator of sulfur metabolism in E. coli.

**TABLE 1:** Differentially expressed genes between E. coli W3110 and Δ*glyA*

Gene name	Expression level		log_2_FC	*P* value	*q* value	Tendency
	W3110	Δ*glyA*				
*glyA*	594.7281	3.4385	−7.4343	0.0000	0.0000	Down
*flu*	411.8295	57.5400	−2.8394	0.0002	0.0062	Down
*cbl*	7.6726	20.6405	1.4277	0.0001	0.0035	Up
*iscR*	296.8332	814.2254	1.4558	0.0000	0.0001	Up
*cysK*	507.0357	1,725.7782	1.7671	0.0000	0.0000	Up
*cysN*	20.4097	135.7450	2.7336	0.0000	0.0000	Up
*cysH*	38.9700	271.4150	2.8001	0.0000	0.0018	Up
*cysA*	31.0602	221.0488	2.8312	0.0000	0.0001	Up
*cysU*	10.9478	88.8963	3.0215	0.0000	0.0000	Up
*cysW*	13.6708	112.2300	3.0373	0.0000	0.0000	Up
*cysI*	39.7372	343.2688	3.1108	0.0000	0.0000	Up
*ydjN*	40.5385	360.8272	3.1539	0.0000	0.0000	Up
*cysD*	18.6454	191.3213	3.3591	0.0000	0.0000	Up
*cysJ*	31.8218	391.4096	3.6206	0.0000	0.0000	Up
*cysP*	24.8206	338.7790	3.7707	0.0000	0.0000	Up
*norV*	51.6395	1,049.8818	4.3456	0.0006	0.0115	Up
*norW*	12.6944	487.3694	5.2628	0.0000	0.0004	Up

### Deletion of *glyA* in E. coli W3110 led to increased susceptibility to novobiocin.

Because *cysB* was shown to be related to NOV susceptibility in E. coli (29), we tested the effect of *glyA* deletion on NOV susceptibility. Our results showed that deletion of *glyA* caused increased sensitivity to NOV (an 8-fold decrease in MIC compared with that for the wild-type strain) (Table 2), which could be fully complemented by introducing an intact copy of *glyA* or high concentrations of exogenous glycine (≥100 μg mL^−1^) (Tables 3 and 4). Although 50 μg mL^−1^ exogenous glycine could support the growth of a *glyA* deletion mutant on E minimal medium, it could only partially complement the NOV-sensitive phenotype. These data suggested that the NOV-sensitive phenotype of Δ*glyA* was caused by glycine deficiency.

**TABLE 2:** MICs of different antimicrobial drugs for E. coli W3110 and E. coli W3110 Δ*glyA*

Drug^*a*^	MIC (μg/mL)	
	E. coli W3110	E. coli W3110 Δ*glyA*
AMP	16	8
KAN	5	5
RIF	8	8
SM	8	4
OFL	0.08	0.04
PE	0.32	0.32
NOV	640	80

a AMP, ampicillin; KAN, kanamycin; RIF, rifampin; SM, streptomycin; OFL, ofloxacin; PE, polymyxin E; NOV, novobiocin.

**TABLE 3:** MICs of NOV of different strains derived from E. coli W3110

Strain	MIC of NOV (μg mL^−1^)
E. coli W3110 pCA24N	640
E. coli W3110 pCA24N::*glyA*	640
E. coli W3110 Δ*glyA* pCA24N	80
E. coli W3110 Δ*glyA* pCA24N::*glyA*	640
E. coli W3110	640
N-15	1,280
E. coli W3110 pCA24N::*yrdC*	640
N-15 pCA24N	640
N-15 pCA24N::*yrdC*	80
E. coli W3110 Δ*glyA*	80
E. coli W3110 Δ*cysB*	1,280
E. coli W3110 Δ*hslJ*	640
E. coli W3110 Δ*gshA*	640
E. coli W3110 Δ*tdcB*	640
E. coli W3110 Δ*cysB* pCA24N	1,280
E. coli W3110 Δ*cysB* pCA24N::*cysB*	640
E. coli W3110 Δ*cysB* pCA24N::*tcyP*	640
E. coli W3110 Δ*glyA*Δ*cysB*	320
E. coli W3110 Δ*glyA*Δ*hslJ*	80
E. coli W3110 Δ*glyA*Δ*tcyP*	640
E. coli W3110 Δ*glyA*Δ*gshA*	640
E. coli W3110 Δ*glyA*Δ*tdcB*	640

**TABLE 4:** MICs of NOV for E. coli W3110 and E. coli W3110 Δ*glyA* in the presence of different concentrations of glycine

Drug(s)	MIC (μg/mL) for E. coli strain	
	W3110	W3110 Δ*glyA*
NOV	640	80
NOV + 10 μg/mL glycine	640	80
NOV + 50 μg/mL glycine	640	160
NOV + 150 μg/mL glycine	640	640

### A reverse mutant of E. coli W3110 Δ*glyA* also had mutation of *yrdC*.

To probe the mechanism by which *glyA* deletion made E. coli W3110 more sensitive to NOV, 14 reverse mutants of Δ*glyA* were selected and their susceptibility to NOV was examined (Table 5). Subsequent genome sequencing of these mutants showed that one of them (N-15) had a 12-bp deletion at the very N terminus of the threonylcarbamoyl-AMP synthase-coding gene *yrdC*. The results of drug susceptibility testing showed that the NOV MIC for N-15 was 640 μg mL^−1^, the same as that for E. coli W3110. By introducing an intact copy of *yrdC* into N-15, the NOV MIC of the complemented strain decreased to 80 μg mL^−1^, the same as that of a Δ*glyA* strain (Table 3).

**TABLE 5:** Characterizations of various reverse mutants of E. coli W3110 Δ*glyA*

Strain	MIC of NOV (μg/mL)	Mutation site
E. coli W3110	640	—^*a*^
E. coli W3110 Δ*glyA*	80	—
N-8	320	—
N-10	640	—
N-12	640	—
N-13	640	—
N-14	640	—
N-15	640	*yrdC*: the 12 bp at the front end of the coding region is missing
N-16	640	—
N-17	640	—
N-18	640	—
N-19	640	—
N-20	640	—
N-21	640	—
N-22	640	—
N-23	640	—

a —, not tested or no useful message found.

### Mutation of *yrdC* affected the expression levels of proteins involved in threonine and glycine metabolism.

**FIG 4:**
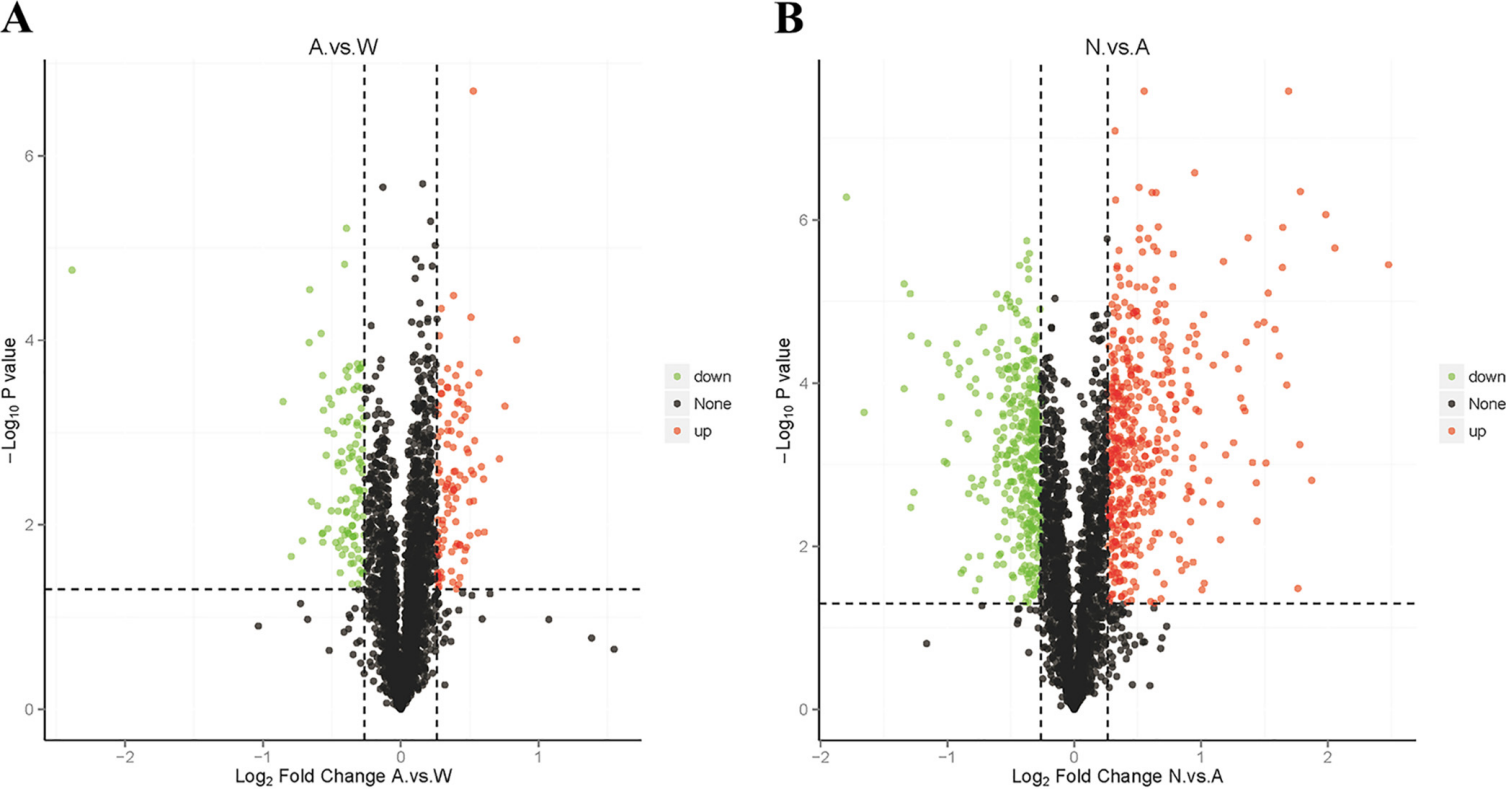
Volcano diagram of differentially expressed proteins. (A) Differentially expressed proteins between E. coli W3110 and E. coli W3110 Δ*glyA*. (B) Differentially expressed proteins between E. coli W3110 Δ*glyA* and N-15. The abscissa represents the difference multiple (log_2_ value) of the differential protein, the vertical axis represents the *P* value (−log_10_ value), the black represents the protein with insignificant difference, the red represents the upregulated protein, and the green represents the downregulated protein. The proteins whose quantitation was significantly different between the experimental and control group (*P* < 0.05 and FC ≥1.2 or FC ≤ 0.83) were defined as differentially expressed proteins.

To further understand why mutation of *yrdC* could reverse the NOV-sensitive phenotype of Δ*glyA*, comparative proteomic analysis was performed. The protein quantitation results were statistically analyzed by two independent-sample *t* tests. The proteins whose quantitation was significantly different between the experimental and control groups (*P* < 0.05 and FC ≥ 1.2 or FC ≤ 0.83) were defined as differentially expressed proteins (Fig. 4). The PCA map of proteomics is provided in Fig. S2. The results showed that, compared with E. coli W3110, the expression levels of three proteins involved threonine degradation (TdcB, TdcE, and TdcF) and TcyP increased in Δ*glyA* mutants (Table 6). However, compared with Δ*glyA*, the expression levels of YrdC and TdcB decreased in N-15, whereas the expression levels of ThrA/B/C and LtaE increased.

**TABLE 6:** Differentially expressed proteins between E. coli W3110, and E. coli W3110 Δ*glyA*, and N-15

Protein name	Description	W3110 Δ*glyA* vs W3110		N-15 vs W3110 Δ*glyA*	
		FC	Tendency	FC	Tendency
TomB	Hha toxicity modulator TomB	1.4	Up	0.65	Down
PrmB	50S ribosomal protein L3 N(5)-glutamine methyltransferase	1.3	Up	0.76	Down
TdcB	Bifunctional threonine ammonia-lyase/l-serine ammonia-lyase TdcB	1.6	Up	0.57	Down
TdcE	2-Ketobutyrate formate-lyase/pyruvate formate-lyase	1.3	Up	0.66	Down
DsdA	d-Serine dehydratase	1.4	Up	0.50	Down
CarB	Carbamoyl-phosphate synthase large chain	1.8	Up	0.41	Down
CarA	Carbamoyl-phosphate synthase small chain	1.4	Up	0.51	Down
YhbW	Luciferase-like monooxygenase	1.5	Up	0.62	Down
GhoS	Endoribonuclease antitoxin GhoS	1.3	Up	0.69	Down
TdcF	Putative reactive intermediate deaminase TdcF	1.2	Up	0.66	Down
ArgE	Acetylornithine deacetylase	1.3	Up	0.78	Down
MalS	Periplasmic alpha-amylase	1.3	Up	0.79	Down
RecX	Regulatory protein RecX	1.4	Up	0.76	Down
NrdF	Ribonucleoside-diphosphate reductase 2 subunit beta	1.5	Up	0.71	Down
TcyP	l-cystine transporter TcyP	1.3	Up	2.28	Up
LeuA	2-Isopropylmalate synthase	NA^*a*^	NA	3.07	Up
LeuB	3-Isopropylmalate dehydrogenase	NA	NA	3.12	Up
LeuC	3-Isopropylmalate dehydratase large subunit	NA	NA	3.66	Up
LeuD	3-Isopropylmalate dehydratase small subunit	NA	NA	3.20	Up
LtaE	Low specificity l-threonine aldolase	NA	NA	1.22	Up
ThrA	Bifunctional aspartokinase/homoserine dehydrogenase 1	NA	NA	3.95	Up
ThrB	Homoserine kinase	NA	NA	4.16	Up
ThrC	Threonine synthase	NA	NA	5.57	Up

a NA, not applicable (no significant difference).

### Deletion of *cysB* partially reversed the NOV-sensitive phenotype of Δ*glyA*, while deletion of *hslJ* did not.

Our comparative proteomic data showed that the expression levels of several proteins involved in sulfur metabolism significantly increased in a *glyA* deletion mutant compared with a wild-type strain, implying that this strain had increased CysB activity; CysB is the major transcriptional regulator of sulfur metabolism in E. coli. Deletion of *cysB* in E. coli W3110 caused a 2-fold increase in the MIC for NOV, and further deletion of this gene in E. coli W3110 Δ*glyA* led to a 4-fold increase in the MIC for NOV (Table 3). This increased resistance to NOV caused by *cysB* deletion could be reversed by overexpressing *tcyP*. Since the *cysB-*regulated gene *hslJ* has been shown to be related to NOV susceptibility in E. coli (29), it was worthwhile to examine whether *hslJ* is also related to the NOV-sensitive phenotype of E. coli W3110 Δ*glyA*. However, deleting *hslJ* in E. coli W3110 did not affect NOV susceptibility; furthermore, deleting this gene in an E. coli W3110 Δ*glyA* strain did not reverse this NOV-sensitive phenotype (Table 3).

### Deletion of *tdcB*, *tcyP*, or *gshA* could reverse the NOV-sensitive phenotype of Δ*glyA*.

Based on our comparative proteomic analysis data, we next deleted *tdcB*, *tcyP*, and *gshA* in E. coli W3110 Δ*glyA*. The results of NOV susceptibility tests showed that further deletion of any of these three genes could reverse the NOV-sensitive phenotype of E. coli W3110 Δ*glyA* (Table 3), but single deletion of these three genes in E. coli W3110 did not affect susceptibility to NOV.

### Deletion of *cycA* in a Δ*glyA* background caused a severe growth defect on LB medium.

**FIG 5:**
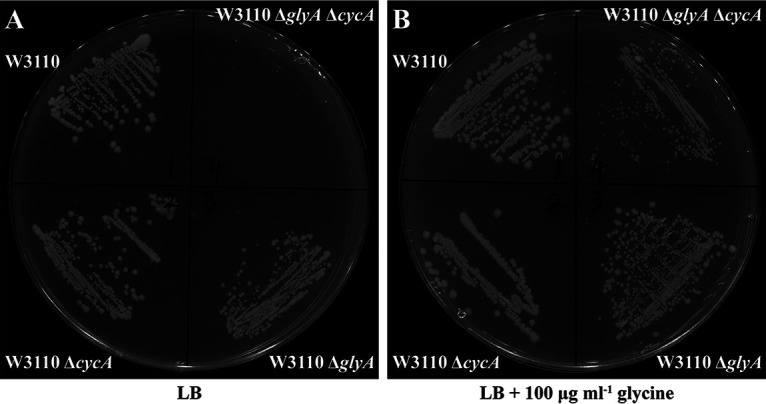
Effect of *cycA* on glycine assimilation in the absence of *glyA.* Growth of E. coli W3110, E. coli W3110 Δ*glyA*, E. coli W3110 Δ*cycA*, and E. coli W3110 Δ*glyA*Δ*cycA* on LB solid medium in the absence of glycine (A) or in the presence of 100 μg mL^−1^ glycine (B). The plates were incubated at 37°C for 18 h.

To test the role of CycA on glycine assimilation in a Δ*glyA* background, a Δ*glyA*Δ*cycA* double-knockout mutant strain was constructed. As shown in Fig. 5, although Δ*glyA* and Δ*cycA* single-deletion mutants grew normally on LB plates, the Δ*glyA*Δ*cycA* strain grew very poorly on LB plates, suggesting that *cycA* plays an important role in glycine assimilation in the absence of *glyA*.

### Overexpression of *cycA* resulted in increased sensitivity to NOV, whereas deleting it caused NOV resistance.

To further test the effect of *cycA* on NOV susceptibility in E. coli W3110, this gene was overexpressed and also knocked out in the strain background. The results showed that overexpressing *cycA* led to increased sensitivity to NOV, while deletion of *cycA* caused NOV resistance (Table 7). However, deleting *cycA* in the E. coli W3110 background did not affect susceptibility to ampicillin (the MIC of ampicillin for the Δ*cycA* mutant was determined to be 32 μg mL^−1^, the same as that for W3110).

**TABLE 7:** MICs of NOV for *cycA*-related strains

Strain	MIC (μg mL^−1^)	
	NOV	NOV + glycine^*a*^
E. coli W3110 pCA24N	640	640
E. coli W3110 Δ*cycA* pCA24N	1,280	1,280
E. coli W3110 Δ*cycA* pCA24N::*cycA*	160	640
E. coli W3110 pCA24N::*cycA*	160	640
E. coli BW25113	1,280	1,280
E. coli BW25113 Δ*glyA*	320	1,280

a The glycine concentration, when added, was 100 μg/mL.

### Overexpression of *cycA* resulted in increased accumulation of NOV upon drug treatment.

**FIG 6:**
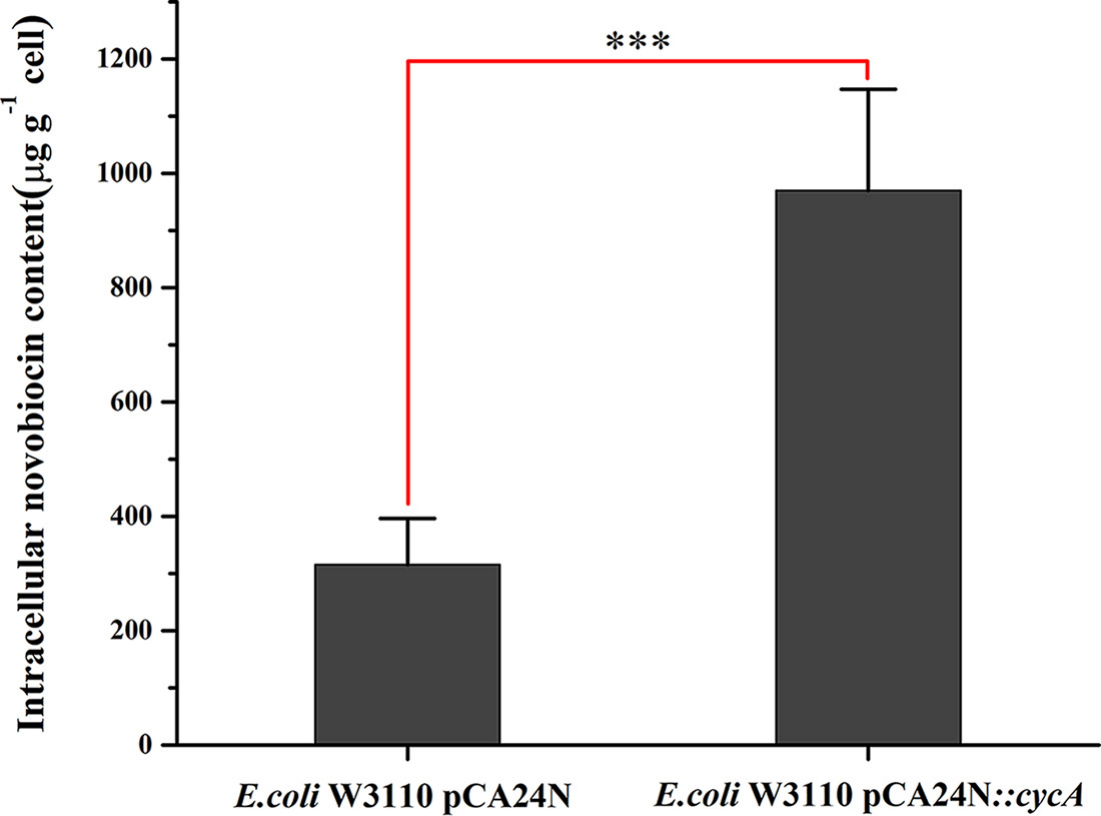
Intracellular contents of NOV in E. coli W3110 pCA24N and E. coli W3110 pCA24N::*cycA*. Bacterial cultures (OD_600_, 0.1) were treated with 640 μg mL^−1^ NOV at 37°C for 2 h. Then, bacterial cells were collected by centrifugation and washed once with PBS precooled at 4°C. The intracellular contents of NOV was detected by HPLC-MS/MS. Data represent mean ± SD from five independent experiments. *P* values were calculated using a two-independent-samples *t* test. ***, significant difference, *P < *0.001.

To explore how CycA affected sensitivity to NOV, we compared the intracellular content of NOV between E. coli W3110 and the *cycA* overexpression strain when treated with this drug. The results showed that the intracellular concentration of NOV in the *cycA* overexpression strain was significantly higher (~3-fold) than that in a wild-type strain (Fig. 6; Table S1).

## DISCUSSION

Although NOV had been used in the clinic for several decades, its mechanisms of susceptibility and resistance are not fully understood. In this study, a linkage between glycine assimilation and NOV susceptibility was established in E. coli.

In an attempt to probe the adaptation of E. coli W3110 in the absence of GlyA, we analyzed comparative transcriptomic data and found evidence for the activation of CysB, the major regulator of sulfur metabolism, which has been shown to be related to susceptibility to NOV in E. coli (30). Moreover, our data from NOV susceptibility testing showed that deleting *glyA* did make E. coli W3110 more sensitive to NOV. However, although CysB did affect the susceptibility of E. coli W3110 to NOV, activation of this protein was not fully responsible for the NOV-sensitive phenotype in E. coli Δ*glyA*, since further deleting *cysB* could only partially reverse this phenotype. Besides, this NOV-sensitive phenotype was not related to *hslJ*, which has been shown to be regulated by CysB and is also involved in NOV susceptibility in E. coli (29, 31).

GlyA is a protein with dual enzymatic activities, one for synthesizing glycine and the other for producing 5,10-methylene-tetrahydrofolate. Since the NOV-sensitive phenotype could be fully reversed by adding exogenous glycine into the growth medium, the glycine-synthesizing activity was responsible for this phenotype, suggesting a linkage between intracellular glycine levels and NOV susceptibility in E. coli.

To further probe the mechanism of the NOV-sensitive phenotype caused by *glyA* deletion, reverse mutants of Δ*glyA* were selected and sent for genome sequencing. Unexpectedly, one of those mutants, N-15, had a 12-bp deletion at the very N terminus of the *yrdC* gene, which encodes a threonylcarbamoyl-AMP synthase, an enzyme involved in translational fidelity in E. coli (32). Subsequent comparative proteomic analysis revealed that deleting *glyA* caused increased expression of TdcB and TcyP. TdcB is the first enzyme of a threonine-degrading pathway that converts threonine into 2-oxobutanoate (33), thus competing with LtaE for threonine utilization. Increased expression of TdcB might cause increased conversion of threonine to 2-oxobutanoate and decreased conversion of threonine to glycine. In E. coli, conversion of threonine into glycine is one of the ways that bacterium produces glycine, although this is not able to fulfill the demands of glycine utilization in the absence of *glyA*. TcyP can also affect glycine abundance within the bacteria. Cystine acquisition is usually followed by its reduction, which accompanies glutathione consumption. Moreover, synthesis of glutathione is an important way of consuming glycine (6, 7). Thus, increased expression of TcyP can lead to increased consumption of glutathione, which in turn causes increased consumption of glycine. However, when comparing the protein expression profiles of Δ*glyA* and N-15, we observed that mutation of *yrdC* caused decreased expression of TdcB but increased expression of ThrA/B/C and LtaE, indicating that more threonine could be synthesized and then converted into glycine. As expected, further deleting *tdcB*, *tcyP*, or *gshA* could completely reverse the NOV-sensitive phenotype of Δ*glyA.* Furthermore, deleting *tdcB* in Δ*glyA* blocked the degradation of threonine into 2-oxobutanoate and increased the flux of threonine into the glycine production reaction catalyzed by LtaE. Additionally, deleting *tcyP* in Δ*glyA* mutants blocked the assimilation of exogenous cysteine, which then decreased the consumption of glutathione and in turn decreased the consumption of glycine. Deleting *gshA* in the Δ*glyA* mutant directly decreased the consumption of glycine. Although our data showed that YrdC could modulate the metabolic efflux of threonine in E. coli, further studies are required to reveal how YrdC achieves this.

Our data revealed that when *glyA* was deleted in E. coli K-12 strains such as W3110 and BW25113, other pathways used for glycine synthesis were also impaired, while glycine consumption was increased. Thus, the bacterium has to rely on the glycine transporter for assimilating exogenous glycine to support its growth. CycA is one of those glycine transporters in E. coli. To test the role of CycA for glycine assimilation in the absence of *glyA*, the *cycA* gene was deleted in Δ*glyA*, and a Δ*glyA*Δ*cycA* mutant showed a severe growth defect on LB medium compared with Δ*glyA* and Δ*cycA* single mutants, suggesting the dependence of CycA for glycine assimilation in the absence of GlyA. However, when high concentrations of exogenous glycine were supplemented into growth medium, the growth defect of the Δ*glyA*Δ*cycA* mutant could be fully reversed, implying the existence of an alternative manner of glycine assimilation which makes CycA unnecessary in the presence of high concentrations of glycine. Deleting *cycA* in E. coli W3110 made bacteria slightly more resistant to NOV, but overexpressing it resulted in increased sensitivity to this drug, showing the linkage between CycA-dependent glycine assimilation and NOV susceptibility in E. coli W3110. As expected, supplementing growth medium with high concentrations of glycine completely reversed the NOV-sensitive phenotype caused by *cycA* overexpression. These data suggested that depending on CycA for glycine assimilation was the cause of increased sensitivity to NOV in the Δ*glyA* mutant.

One of the common factors affecting drug susceptibility is drug accumulation inside a bacterial cell. For better understanding of how CycA-dependent glycine assimilation affects NOV sensitivity in E. coli, the accumulation of NOV was compared between the *cycA* overexpression strain and E. coli W3110, and the results showed that the amount of NOV accumulated in the *cycA*-overexpressing strain was significantly higher. Since CycA is a glycine transporter, it was less likely that this protein could also transport NOV. Very recently, Gallagher et al. showed that inactivation of *cycA* in Staphylococcus aureus made the bacterium more sensitive to β-lactams, since d-alanine incorporation into cell wall peptidoglycan was impaired (34). However, deleting *cycA* in E. coli W3110 did not affect susceptibility to β-lactams. It would be worthwhile to further explore how a glycine transporter affects the transportation of coumarin family compounds.

**FIG 7:**
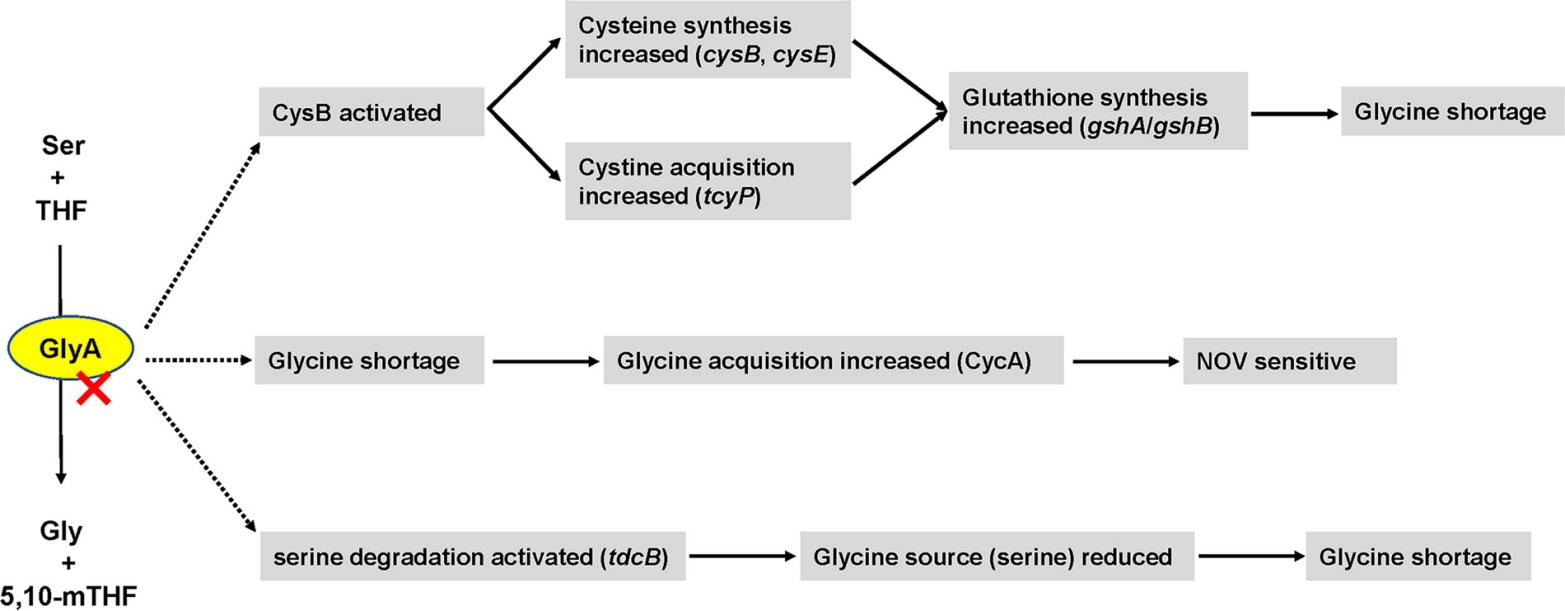
Diagram of changes in metabolic pathways caused by *glyA* mutation in Escherichia coli K-12 W3110. *glyA* mutation resulted in glycine shortage in three ways: (i) increased cysteine and glutathione synthesis by activating CysB to increase glycine consumption; (ii) lack of glycine synthesis; (iii) activated serine degradation to reduce the major source of glycine. Glycine shortage caused by *glyA* mutation led to heavy dependence on CycA to acquire glycine from the medium.

In summary, in E. coli W3110, deleting *glyA* impaired glycine biosynthesis and increased the consumption of glycine, thus making bacteria more dependent on CycA for glycine assimilation and hence leading to increased susceptibility to NOV (Fig. 7). To our knowledge, this is the first report demonstrating the linkage between glycine assimilation and NOV susceptibility and also the first report showing that YrdC is able to modulate the metabolic flux of threonine.

## MATERIALS AND METHODS

### Bacterial strains, plasmids, and culture conditions.

Details of all strains and plasmids used in this study are listed in Table S2 in the supplemental material. Strains of E. coli were cultured in LB medium (Difco) or E minimal medium (we dissolved 0.2 g of MgSO_4_·7H_2_O, 2 g of citric H_2_O, 13.09 g of K_2_HPO_4_·3H_2_O, and 3.5 g of NaNH_4_HPO_4_·4H_2_O in 1 liter of deionized water) at 37°C. The plasmids pKD4, pKD46, and pCP20 were used for construction of gene deletion mutants. Plasmid pCA24N (35) was used for gene expression. Gene-specific primers used for the construction of recombinant plasmids are listed in Table S3. Where appropriate, the E. coli culture medium was supplemented with 50 μg mL^−1^ kanamycin, 50 μg mL^−1^ chloramphenicol, or 100 μg mL^−1^ ampicillin.

### Construction of gene deletion mutants, complemented strains, and gene overexpression strains.

Gene knockout mutants in E. coli W3110 were constructed with the λ Red recombination system and verified by junction PCR as previously described (36). Double-knockout mutants were generated with the same procedure. To construct complemented or gene overexpression strains, genes were amplified from E. coli W3110 genomic DNA using gene-specific primers (Table S3) and cloned into pCA24N. These recombinant plasmids were then transformed into either the corresponding knockout mutants (to form the complemented strain) or E. coli W3110 to obtain complemented or gene overexpression strains.

### Drug susceptibility tests.

Bacterial cells were grown to the mid-log phase (optical density at 600 nm [OD_600_] of 0.6 to 1.0) and then diluted to 10^5^ CFU mL^−1^ in fresh LB. Next, 10-fold serial dilutions were plated on LB plates with different concentrations of NOV (0, 10, 20, 40, 80, 160, 320, 640, and 1,280 μg mL^−1^). Then, 20 μM isopropyl-β-d-thiogalactopyranoside was added to the growth medium when complemented and/or gene overexpression strains were tested. Cultures were incubated overnight at 37°C. The MIC was defined as the lowest concentration of NOV required to inhibit 99% of the CFU.

### RNA-seq.

Total RNA was isolated with an RNeasy minikit (Qiagen, Hilden, Germany). Library constructions were prepared using a TruSeq stranded total RNA sample preparation kit (Illumina, San Diego, CA, USA), and RNA was sequenced with an Illumina HiSeq 2500 system at the Shanghai Biotechnology Corporation (Shanghai, China). The median insert sizes of the purified libraries were confirmed with an Agilent 2100 bioanalyzer (Agilent Technologies, Santa Clara, CA, USA). Bowtie2 v2-2.0.5 was used to align the cleaned reads to the Escherichia coli strain K-12 substrain W3110 genome downloaded from the National Center for Biotechnology Information (NCBI) website (https://www.ncbi.nlm.nih.gov/genome/167?genome_assembly_id=161529). The read depth was 23 to 33 M reads. The coverage of the tested samples in comparison to the reference genome was >96%. To estimate fold changes, HTSeq v2.1.1 was used with a reference annotation to generate values for fragments per kilobase of exon model per million mapped reads. Three biological replicates were used for RNA-seq, and *P* and *q* values were calculated. Differentially expressed genes were selected only if the false-discovery rate *q* was ≤0.05 and fold change was ≥2.

### Screening for reverse mutants of Δ*glyA*.

E. coli Δ*glyA* was cultured in LB liquid medium at 37°C to mid-log phase (OD_600_ of 0.6 to 1.0), diluted to 10^8^ CFU mL^−1^ with fresh LB, and then plated on LB plates containing 160 μg mL^−1^ NOV (10^6^ CFU per plate). Plates were incubated overnight at 37°C. Reverse mutants were randomly selected from NOV-containing plates, and the MICs of NOV were then tested. Next, some of these reverse mutants (NOV MICs ≥640 μg mL^−1^) were sent for genome sequencing (SHBIO, Shanghai, China).

### Comparative genomic analysis between Δ*glyA* and reverse mutants.

Bacterial genomic DNA was extracted using commercial kits (New England Biolabs, USA). The extracted DNA was examined by using Qubit 2.0 and 0.8% agarose gel electrophoresis. Genomic libraries were constructed in accordance with the test instructions. Fragment libraries were sequenced on an Illumina HiSeq with double-ended sequencing. The sequencing process was controlled by the data collection software provided by Illumina, and real-time data analysis was performed. Routine genome analysis was performed on the whole-genome sequencing data of the sample, including data preprocessing, genome mapping, single-nucleotide polymorphism detection and annotation, InDel detection and annotation, and structural variant detection and annotation based on the reads comparison. The read depth was ×725 with 13 to 19 M reads. The coverage of the tested samples compared to the reference genome was >99%.

### Comparative proteomic analysis between E. coli W3110, Δ*glyA*, and N-15.

**(i) Total protein extraction.** Samples were transferred to 1.5-mL centrifuge tubes and lysed with SDT lysis buffer (4% SDS, 10 mM dithiothreitol [DTT], and 100 mM trimethylammonium bicarbonate [TEAB]), followed by 5 min of ultrasonication on ice. The lysate was centrifuged at 12,000 × *g* for 15 min at 4°C, and the supernatant was reduced with 10 mM DTT for 1 h at 56°C and subsequently alkylated with sufficient iodoacetamide for 1 h at room temperature in the dark. Then, samples were completely mixed with 4 volumes of precooled acetone by vortexing and then incubated at −20°C for at least 2 h. Samples were then centrifuged at 12,000 × *g* for 15 min at 4°C and the precipitate was collected. After washing with 1 mL of cold acetone, the pellet was dissolved in dissolution buffer (8 M urea, 100 mM TEAB; pH 8.5).

**(ii) TMT labeling of peptides.** For each protein sample, the volume was brought up to 100 μL with DB dissolution buffer (8 M urea, 100 mM TEAB; pH 8.5) after protein quality testing. Trypsin and 100 mM TEAB buffer were then added, and each sample was mixed and digested at 37°C for 4 h. Next, trypsin and CaCl_2_ were added, and each sample was digested overnight. Formic acid was mixed with the digested sample, the pH was adjusted to >3, and samples were centrifuged at 12,000 × *g* for 5 min at room temperature. The supernatant was slowly loaded onto a C_18_ desalting column and washed with washing buffer (0.1% formic acid and 3% acetonitrile) three times, and then samples were eluted with elution buffer (0.1% formic acid and 70% acetonitrile). The eluents of each sample were collected and subsequently lyophilized; 100 μL of 0.1 M TEAB buffer was added to reconstitute these samples, 41 μL of acetonitrile-dissolved tandem mass tag (TMT) labeling reagent was added, and each sample was mixed with shaking for 2 h at room temperature. Then, the reaction was stopped by adding 8% ammonia. All labeled samples were mixed to an equal volume, desalted, and lyophilized until analysis.

**(iii) Separation of fractions.** Mobile phases A (2% acetonitrile, adjusted to a pH 10.0 using ammonium hydroxide) and B (98% acetonitrile) were used to develop a gradient elution. The lyophilized powder was dissolved in solution A and centrifuged at 12,000 × *g* for 10 min at room temperature. These samples were fractionated using a C_18_ column (Waters BEH C_18_; 4.6 by 250 mm, 5 μm) on a Rigol L3000 high-performance liquid chromatography (HPLC) system, and the column oven was set at 45°C. The eluates were monitored by UV at 214 nm, collected at 1 tube per min, and combined into 10 final fractions. All fractions were dried by vacuum and then reconstituted in 0.1% (vol/vol) formic acid in water.

**(iv) LC-MS/MS analysis.** For transition library construction, shotgun proteomics analyses were performed using an EASY-nLC 1200 UHPLC system (Thermo Fisher) coupled with a Q Exactive series mass spectrometer (Thermo Fisher) operating in the data-dependent acquisition mode. One microgram of sample was injected into a home-made C_18_ Nano-Trap column (4.5 cm by 75 μm, 3 μm). Peptides were separated in a home-made analytical column (15 cm by 150 μm, 1.9 μm), using a linear gradient elution (0 min, 94% A; 2 min, 85% A; 87.5 min, 60% A; 80.5 min, 50% A; 81.5 min, 45% A; 90 min, 0% A). The separated peptides were analyzed using a Q Exactive series mass spectrometer (Thermo Fisher) with a Nanospray Flex (electrospray ionization [ESI]) ion source, a spray voltage of 2.3 kV, and an ion transport capillary temperature of 320°C. The full scan range from *m/z* 350 to 1,500, with a resolution of 60,000 (at *m/z* 200), an automatic gain control (AGC) target value of 3 × 10^6^, and a maximum ion injection time of 20 ms was used. The top 40 precursors of the highest abundance in the full scan were selected and fragmented by higher-energy collisional dissociation and analyzed by tandem mass spectrometry (MS/MS), where the resolution was 45,000 (at *m/z* 200) for 10-plex, the AGC target value was 5 × 10^4^, the maximum ion injection time was 86 ms, the normalized collision energy was set at 32%, the intensity threshold was 1.2 × 10^5^, and the dynamic exclusion parameter was 20 s.

**(v) Data analysis.** The resulting spectra from each run were searched separately against the escherichia_coli_uniprot_2020_7_2.fasta (9,432 sequences) database using the search engine Proteome Discoverer 2.2 (PD 2.2; Thermo). The searched parameters were set as follows: the mass tolerance for each precursor ion was 10 ppm, and the mass tolerance for the product ions was 0.02 Da. Carbamidomethyl was specified as a fixed modification, whereas oxidation of methionine (M) and TMT plex were specified as dynamic modifications; acetylation and TMT plex were specified as N-terminal modifications in PD 2.2. A maximum of two miscleavage sites were allowed.

To improve the quality of analysis results, the software PD 2.2 further filtered the retrieval results. Peptide spectrum matches (PSMs) with a credibility of >99% were identified as PSMs. The identified protein contained at least 1 unique peptide. The identified PSMs and proteins were retained and screened for those having a false-discovery rate of ≤1.0%. The protein quantitation results were then statistically analyzed by a two-independent-sample *t* test. The proteins whose quantitation was significantly different between the experimental and control groups (*P* < 0.05 and FC ≥ 1.2 or FC ≤ 0.83) were defined as differentially expressed proteins.

### Measurement of intracellular content of NOV.

Bacterial cells were grown to mid-log phase (OD_600_, 0.5 to 1.0) and diluted to 10^8^ CFU mL^−1^ in 50 mL fresh LB with 640 μg mL^−1^ NOV. Subsequently, these 50-mL bacterial cultures were grown at 37°C with shaking (160 rpm) for 2 h. After that, cell densities (OD_600_) were measured and recorded. Then, bacterial cells were collected by centrifugation (8,000 rpm, 10 min), washed once with phosphate-buffered saline (PBS) precooled at 4°C, and frozen at −80°C until use.

**(i) Extraction of samples.** All samples were accurately weighed in test tubes, 2 mL of a methanol solution was added, and samples were subjected to ultrasonic extraction for 10 min. The supernatant was taken, and samples were blow-dried, redissolved with 0.2 mL 50% methanol, filtered with 0.22-μm organic filter membranes after centrifugation, and then measured on-line (if sample content was too high, it was diluted 100 times before on-line measurement).

**(ii) Establishment of standard curve.** Novobiocin was dissolved in methanol to provide a 1-μg mL^−1^ novobiocin standard solution, which was then diluted into six gradient standard solutions (5, 10, 50, 100, 500, and 1,000 ng mL^−1^). Each gradient standard solution was tested by HPLC-MS. The standard curve was established between the concentrations and the characteristic peak intensities in mass spectra.

**(iii) HPLC-MS/MS protocol.** The novobiocin solutions were analyzed on a 6420A mass spectrometer (Agilent, USA) coupled with a 1260 HPLC system (Agilent, USA). The injected solution (2 μL) was separated on a poroshell 120 SB-C_18_ reversed-phase chromatographic column (2.1 cm by 150 mm, 2.7 μm) at a flow rate of 0.3 mL min^−1^, with the column temperature at 30°C. The separation gradient employed was as follows: 5 min, 5% A; 8 min, 5% A; 8.10 min, 30% A. For the novobiocin target detection, ESI positive and negative ion modes were monitored in a multiple-reaction mode scanning type. Other parameters were as follows: air curtain, 15 lb/in^2^; spray voltage, +4,000 V, −3,500 V; atomization gas pressure, 65 lb/in^2^; auxiliary gas pressure, 70 lb/in^2^; atomization temperature, 350°C.

### Data availability.

The raw transcriptomics analysis data (between E. coli W3110 and E. coli W3110 Δ*glyA*) have been deposited in the NCBI database with the data set identifier PRJNA882203. The raw genomics analysis data (between E. coli W3110 Δ*glyA* and revertants) have been deposited in the NCBI database with the data set identifier PRJNA885173. The mass spectrometry proteomics analysis data have been deposited with the ProteomeXchange Consortium (http://proteomecentral.proteomexchange.org/cgi/GetDataset?ID=PXD037221) via the iProX partner repository with the data set identifier PXD037221. The RNA-seq and DNA-seq data were provided by Shanghai Bohao Biotechnology Co., Ltd. The proteomics data were provided by Novogene Co., Ltd (Nanjing, China).

## Supplementary Material

Reviewer comments
