# Supplementary material for: CycA-Dependent Glycine Assimilation Is Connected to Novobiocin Susceptibility in Escherichia coli
Source: Microbiol Spectr. 2022 Nov 15;10(6):e02501-22. doi: 10.1128/spectrum.02501-22 (PMC9769978; doi:10.1128/spectrum.02501-22)
Supplement: Supplemental file 1 — Supplemental material. Download spectrum.02501-22-s0001.pdf, PDF file, 0.3 MB [file spectrum.02501-22-s0001.pdf]

Table S1 Intracellular contents of NOV in *E. coli* W3110 pCA24N and *E. coli* W3110 pCA24N::*cycA*.

No. of Samples	Strains	Samples		Novobiocin	
		Volume (mL)	Weight (g)	Concentration (ng mL^{-1})	Intracellular content (ug g^{-1})
1	W3110 pCA24N	50.00	0.0476	432.68	454.50
2	W3110 pCA24N	50.00	0.0427	297.96	348.90
3	W3110 pCA24N	50.00	0.0457	251.39	275.04
4	W3110 pCA24N	50.00	0.0454	198.84	218.99
5	W3110 pCA24N	50.00	0.0210	117.20	279.04
6	W3110 pCA24N::*cycA*	50.00	0.0386	815.48	1056.32
7	W3110 pCA24N::*cycA*	50.00	0.0437	949.84	1086.77
8	W3110 pCA24N::*cycA*	50.00	0.0413	519.36	628.76
9	W3110 pCA24N::*cycA*	50.00	0.0274	608.62	1110.62
10	W3110 pCA24N::*cycA*	50.00	0.0402	777.09	966.52

Table S2 strains and plasmids used in this study

Strains/Plasmids	description	Source
**strains**		
E. coli W3110	Wild type *E. coli* strain used in this study	Laboratory stock
E. coli W3110 pCA24N	*E. coli* W3110 with empty plasmid pCA24N	This study
E. coli W3110 Δ*glyA*	*glyA* deletion strain	This study
E. coli W3110 Δ*glyA* pCA24N	*glyA* deletion strain with empty plasmid pCA24N	This study
E. coli W3110 pCA24N::*glyA*	*glyA* overexpression strain	This study
E. coli W31Δ*glyA* pCA24N::*glyA*	*glyA* complemented strain	This study
E. coli W3110 Δ*cycA*	*cycA* deletion strain	This study
E. coli W3110 Δ*cycA* pCA24N	*cycA* deletion strain with empty plasmid pCA24N	This study
E. coli W3110 pCA24N::*cycA*	*cycA* overexpression strain	This study
E. coli W3110 Δ*cycA* pCA24N::*cycA*	*cycA* complemented strain	This study
E. coli W3110 Δ*glyA*Δ*cycA*	*glyA* and *cycA* double deletion strain	This study
E. coli W3110 Δ*cysB*	*cysB* deletion strain	This study
E. coli W3110 Δ*glyA*Δ*cysB*	*glyA* and *cysB* double deletion strain	This study
E. coli W3110 Δ*tcyP*	*tcyP* deletion strain	This study
E. coli W3110 Δ*glyA*Δ*tcyP*	*glyA* and *tcyP* double deletion strain	This study
E. coli W3110 Δ*cysB* pCA24N	*cysB* deletion strain with empty	This study

	plasmid pCA24N	
E. coli W3110 Δ*cysB* pCA24N::*cysB*	*cysB* complemented strain	This study
E. coli W3110 Δ*cysB* pCA24N::*tcyP*	*cysB* deletion strain with *tcyP* overexpression	This study
E. coli W3110 Δ*glyA*Δ*hslJ*	*glyA* and *hslJ* double deletion strain	This study
E. coli W3110 Δ*gshA*	*gshA* deletion strain	This study
E. coli W3110 Δ*glyA*Δ*gshA*	*glyA* and *gshA* double deletion strain	This study
E. coli W3110 Δ*gshB*	*gshB* deletion strain	This study
E. coli W3110 Δ*glyA*Δ*gshB*	*glyA* and *gshB* double deletion strain	This study
E. coli W3110 Δ*tdcB*	*tdcB* deletion strain	This study
E. coli W3110 Δ*glyA*Δ*tdcB*	*glyA* and *tdcB* double deletion strain	This study
N-15	*glyA* deletion strain with *yrdC* reverse mutation	This study
N-15 pCA24N	N-15 with empty plasmid pCA24N	This study
E. coli W3110 pCA24N::*yrdC*	*yrdC* overexpression strain	This study
N-15 pCA24N::*yrdC*	N-15 with *yrdC* overexpression	This study
BL21	The strain used for gene clone	Laboratory stock
S-9	Revertant mutant	This study
S-11	Revertant mutant	This study
S-18	Revertant mutant	This study
N-8	Revertant mutant	This study
N-10	Revertant mutant	This study
N-12	Revertant mutant	This study
N-13	Revertant mutant	This study
N-14	Revertant mutant	This study
N-15	Revertant mutant	This study
N-16	Revertant mutant	This study
N-17	Revertant mutant	This study
N-18	Revertant mutant	This study
N-19	Revertant mutant	This study
N-20	Revertant mutant	This study
N-21	Revertant mutant	This study
N-22	Revertant mutant	This study
N-23	Revertant mutant	This study
**Plasmids**		
pKD4	The plasmid used for genes knockout	Laboratory stock
pKD46	The plasmid used for genes knockout	Laboratory stock

pCP20	The plasmid used for genes knockout	Laboratory stock
pCA24N	The plasmid used for genes overexpression	Laboratory stock
pCA24N::*glyA*	The plasmid used for *glyA* overexpression	This study
pCA24N::*cycA*	The plasmid used for *cycA* overexpression	This study
pCA24N::*cysB*	The plasmid used for *cysB* overexpression	This study
pCA24N::*tcyP*	The plasmid used for *tcyP* overexpression	This study
pCA24N::*yrdC*	The plasmid used for *yrdC* overexpression	This study

Table S3 primers used in this study

Primers name	Sequence (5´-3´)
glyA-KFP	ATGTTAAAGCGTGAAATGAACATTGCCGATTATGATGCCGAACTGTGGCAATATGAATATCCTCCTTAG
glyA-KRP	TTATGCGTAAACCGGGTAACGTGCGCAGATGTCGAGAACTTTACCTTTGATGTAGGCTGGAGCTGCTTCG
glyA-CFP	ATGAACAACGAGCACATTGACAGC
glyA-CRP	TCCAAAGCCTTGCGTAGCCTGA
glyA-*Sac*I-FP	ATAGAGCTCATGTTAAAGCGTG
glyA-*Not*I-RP	ATTTGCGGCCGCTTATGCGTAAACC
cycA-KFP	ATGGTAGATCAGGTAAAAGTCGTTGCCGATGATCAGGCTCCGGCTGAACAATATGAATATCCTCCTTAG
cycA-KRP	TTATTTCCGCAGTTCAGCAGCCCGCTTCTTACCAATAAACAGCCAGCCCATGTAGGCTGGAGCTGCTTCG
cycA-CFP	ACACAGACAGGTACAGGAAGAA
cycA-CRP	CAGCCACATGATGAAAGAAGAG
cycA-*Sac*I-FP	TTAAGAGCTCATGGTAGATCAGGTAAAAGTCG
cycA-*Not*I-RP	TATTGCGGCCGCTTATTTCCGCAGTTCAGCAGCC
cysB-KFP	ATGAAATTACAACAACTTCGCTATATTGTTGAGGTGGTCAATCATAACCT ATATGAATATCCTCCTTAG
cysB-KRP	TTATTTTTCCGGCAGTTTTATATCTTTAAACATGACCTCAATTTCTTCAT ATATGAATATCCTCCTTAG
cysB-CFP	ACGATGTTCTGATGGCGTCTA
cysB-CRP	CAAAAATCCAGCGAATTTACG
cysB-*Bgl*II-FP	AATTAGATCTATGAAATTACAACAACTTCGC
cysB-*Xba*I-RP	AATTTCTAGATTATTTTTCCGGCAGTTTT
tcyP-KFP	ATGAACTTTCCATTAATTGCGAACATCGTGGTGTTCGTTGTACTGCTGTTATATGAATATCCTCCTTAG

tcyP-KRP	TTAATGGTGTGCCAGTTCGGCGTCGTCTTCGCTATCC<br>AGAATGGCTTTATTGTAGGCTGGAGCTGCTTCG
tcyP-CFP	TAATAAGATCAGGAGAACGGGG
tcyP-CRP	GGAACATTTAAAAAGTAAGGCA
*tcyP-Bgl*II-FP	TATAAGATCTATGAACTTTCCATTAATTGCGAAC
*tcyP-Sac*I-RP	AATTGAGCTCTTAATGGTGTGCCAGTTCGGC
gshA-KFP	TTGATCCCGGACGTATCACAGGCGCTGGCCTGGCTGG<br>AAAAACATCCTCAATATGAATATCCTCCTTAG
gshA-KRP	TCAGGCGTGTTTTTCCAGCCACACCGCAAACGGTTCG<br>GTATCAGCGGCTTTGTAGGCTGGAGCTGCTTCG
gshA-CFP	TAATCTTTTTTTTGTGCCTTGG
gshA-CRP	CGCATTTGTTATCATCATCCCT
gshB-KFP	ATGATCAAGCTCGGCATCGTGATGGACCCCATCGCAA<br>ACATCAACATCAAATATGAATATCCTCCTTAG
gshB-KRP	TTACTGCTGCTGTAAACGTGCTTCGATGGCATCCATT<br>AACATTCCGGTGATGTAGGCTGGAGCTGCTTCG
gshB-CFP	GTGTGCAGAGCAGGATGAAGGA
gshB-CRP	AAATGTAGACCACGGAACGACG
tdcB-KFP	ATGCATATTACATACGATCTGCCGGTTGCTATTGATG<br>ACATTATTGAAGCATATGAATATCCTCCTTAG
tdcB-KRP	TTAAGCGTCAACGAAACCGGTGATTTGAGAGACGCG<br>AGAAAGATCGATATTGTAGGCTGGAGCTGCTTCG
tdcB-CFP	GCAGGTGTCGGTTACGGTTACC
tdcB-CRP	ACGCCAGGACGATTGTTTTGTC
yrdC-Sac1-FP	TAATGAGCTCATGAATAATAACCTGCAAAGAGACG
yrdC-Not1-RP	AATTGCGGCCGCTTACCCCTGTCGAAACAGTTCAC

Note: gene name + K, primers designed for knocking-out of the gene; Gene name + C, primers used for verification of the gene knockout mutant; Gene name + FP, upstream primers of the gene; Gene name + RP, the downstream primers of the gene; gene name + restriction enzyme name, primers used for overexpression of the gene; The underlined part of all primers used for constructing the gene knockout mutants is the homologous arm of the target gene.

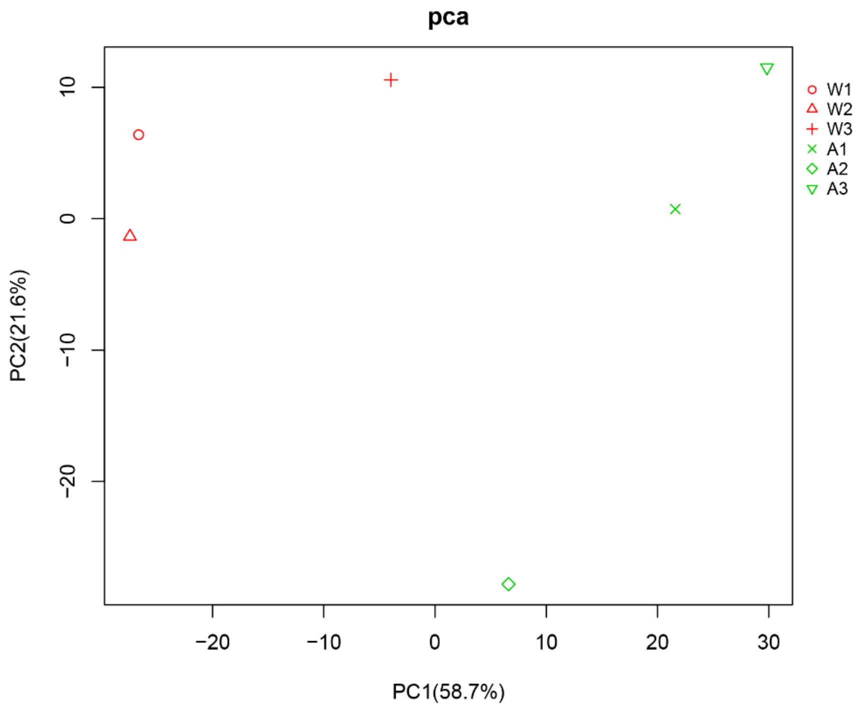

Figure S1 PCA map of RNA-seq based on gene expression. Principal component analyses were conducted for RNA-seq between *E. coli* W3110 (W1-3) and *E. coli* W3110 Δ*glyA* (A1-3).

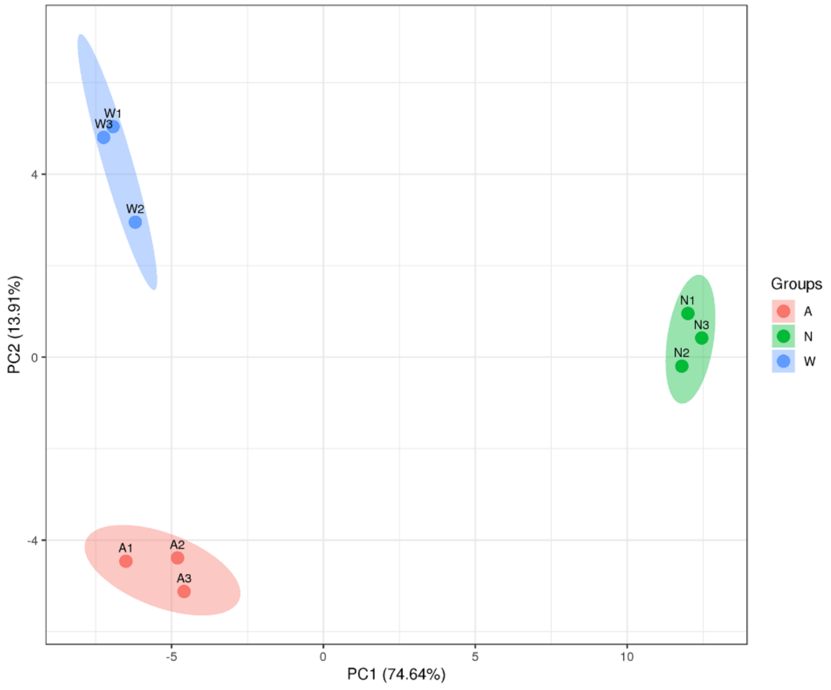

Figure S2 PCA map of proteomics based on protein expression. Principal component analyses were conducted for proteomics among *E. coli* W3110 (W), *E. coli* W3110 Δ*glyA* (A) and N-15 (N).
