# Supplementary material for: CycA-Dependent Glycine Assimilation Is Connected to Novobiocin Susceptibility in Escherichia coli
Source: Microbiol Spectr. 2022 Nov 15;10(6):e02501-22. doi: 10.1128/spectrum.02501-22 (PMC9769978; doi:10.1128/spectrum.02501-22)
Supplement: Reviewer comments [file reviewer-comments.pdf]

AMERICAN SOCIETY FOR MICROBIOLOGY | Microbiology Spectrum

CycA-dependent glycine assimilation is connected to novobiocin susceptibility in Escherichia coli

Hongmei shi, Ling Zhang, Jing Gu, Jinyue Li, Zixiang Liu, and Jiaoyu Deng

Corresponding Author(s): Jiaoyu Deng, Key Laboratory of Special Pathogens and Biosafety, Wuhan Institute of Virology, Chinese Academy of Sciences

Review Timeline:

Submission Date:	July 7, 2022
Editorial Decision:	September 8, 2022
Revision Received:	October 30, 2022
Accepted:	November 3, 2022

Editor: Jeffrey Gralnick

Reviewer(s): The reviewers have opted to remain anonymous.

Transaction Report:

(Note: With the exception of the correction of typographical or spelling errors that could be a source of ambiguity, letters and reports are not edited. The original formatting of letters and referee reports may not be reflected in this compilation.)

DOI: https://doi.org/10.1128/spectrum.02501-22

September 8, 2022

Prof. Jiaoyu Deng
Key Laboratory of Special Pathogens and Biosafety, Wuhan Institute of Virology, Chinese Academy of Sciences
middle street of xiaohongshan, NO.44
Wuhan, Hubei 430071
China

Re: Spectrum02501-22 (CycA-dependent glycine assimilation connected to novobiocin susceptibility in *Escherichia coli*)

Dear Prof. Jiaoyu Deng:

Thank you for submitting your manuscript to Microbiology Spectrum. Rather than continue to wait for a second review, I think it is best to return the manuscript with comments only from Reviewer 1, who has provided a thorough review of your manuscript. When submitting the revised version of your paper, please provide (1) point-by-point responses to the issues raised by the reviewers as file type "Response to Reviewers," not in your cover letter, and (2) a PDF file that indicates the changes from the original submission (by highlighting or underlining the changes) as file type "Marked Up Manuscript - For Review Only". Please use this link to submit your revised manuscript - we strongly recommend that you submit your paper within the next 60 days or reach out to me. Detailed instructions on submitting your revised paper are below.

Link Not Available

Below you will find instructions from the Microbiology Spectrum editorial office and comments generated during the review.

ASM policy requires that data be available to the public upon online posting of the article, so please verify all links to sequence records, if present, and make sure that each number retrieves the full record of the data. If a new accession number is not linked or a link is broken, provide production staff with the correct URL for the record. If the accession numbers for new data are not publicly accessible before the expected online posting of the article, publication of your article may be delayed; please contact the ASM production staff immediately with the expected release date.

The ASM Journals program strives for constant improvement in our submission and publication process. Please tell us how we can improve your experience by taking this quick Author Survey.

Sincerely,

Jeffrey Gralnick

Senior Editor, Microbiology Spectrum

Journals Department
American Society for Microbiology
1752 N St., NW
Washington, DC 20036
E-mail: spectrum@asmusa.org

Editor comments:

Please note that ASM Journals have an open data policy and requires a 'Data Availability Statement' that provides information where readers (and reviewers) can access data (e.g. RNA-seq, proteomics etc). More information can be found here: https://journals.asm.org/open-data-policy

Reviewer comments:

Reviewer #1 (Comments for the Author):

In this manuscript, the authors explore an association between glycine metabolism and novobiocin susceptibility in E. coli. While novobiocin is not currently used as an antimicrobial agent and is not viewed as effective against Gram negative pathogens, drug

repurposing efforts merit exploration of orphan drug activity in novel contexts. In this study, the authors use a combination of transcriptomics, proteomics, site-directed mutagenesis and mass-spectrometry to determine that the basis for lack of efficacy of novobiocin against E. coli is related to limited uptake of the drug across the inner-membrane. This finding alone is novel as it is generally thought that the barrier to activity is, in part, through outer-membrane mediated exclusion. Evidence is presented that suggests transport is associated with glycine acquisition, can be modulated by glycine metabolism and is facilitated by the major glycine transporter CycA. While this is a complex story, it is presented in a way that is somewhat easy to follow.

Major comments:
Since this story is quite complex, with numerous relevant intersecting metabolic pathways, regulators and transporters, it would be highly beneficial to include a figure that assimilates all of the key points that the authors are looking to convey.

Tables in the manuscript have repetitive information and can be highly condensed. Tables 3, 6 & 8 should be combined. Table 5 can be eliminated as it does not provide any information that is not found elsewhere in the manuscript.

The authors have used RNA-seq and proteomics to test and generate key ideas within this manuscript. However, very little of these data are shown. It is recommended that the authors include some representation of these global datasets, such as volcano plots. It is also essential that the authors perform and present more rigorous analysis of these data, for example through the use of principal component analyses.

There is insufficient description of read depth or thresholds for read quality in RNA-seq and full genome sequencing.

For mass spec results, there is no description of extraction controls nor methods and standards for quantification, which are essential for quantitative MS from cellular extracts. Raw data should be provided as supplemental information.

Figure legends are not sufficiently informative. Please add enough information in the figure legends for readers to understand what the data represent. For Figure 3, provide additional information regarding statistical analysis, "P values were calculated using t tests" is not adequate. There are numerous versions of and approaches for t tests.

Minor points:
Line 69, start new paragraph at "CycA is an inner membrane protein..."
Line 89, change "reports" to "report"
Lines 90, 106 and 259, change "minimum" to "minimal"
Line 100, need a citation for "Since cysB was shown to be related to NOV susceptibility in E.coli..."
Lines 127, 128, 139 and 146, delete "Further"
Line 136, provide some rationale for why hslJ was examined
Line 201, change "level of" to "abundance within the"
Line 202, change "assimilation" to "acquisition"
Line 229, change "recovered" to "reversed"
Line 367, there is incorrect reference to Table 2, there is no table provided that gives the described information
Line 379, it is not clear what is meant by "*** database"

Staff Comments:

Preparing Revision Guidelines
To submit your modified manuscript, log onto the eJP submission site at https://spectrum.msubmit.net/cgi-bin/main.plex. Go to Author Tasks and click the appropriate manuscript title to begin the revision process. The information that you entered when you first submitted the paper will be displayed. Please update the information as necessary. Here are a few examples of required updates that authors must address:

• Point-by-point responses to the issues raised by the reviewers in a file named "Response to Reviewers," NOT IN YOUR COVER LETTER.
• Upload a compare copy of the manuscript (without figures) as a "Marked-Up Manuscript" file.
• Each figure must be uploaded as a separate file, and any multipanel figures must be assembled into one file.
• Manuscript: A .DOC version of the revised manuscript
• Figures: Editable, high-resolution, individual figure files are required at revision, TIFF or EPS files are preferred

For complete guidelines on revision requirements, please see the journal Submission and Review Process requirements at https://journals.asm.org/journal/Spectrum/submission-review-process. **Submissions of a paper that does not conform to**

Microbiology Spectrum guidelines will delay acceptance of your manuscript. "

Please return the manuscript within 60 days; if you cannot complete the modification within this time period, please contact me. If you do not wish to modify the manuscript and prefer to submit it to another journal, please notify me of your decision immediately so that the manuscript may be formally withdrawn from consideration by Microbiology Spectrum.

If your manuscript is accepted for publication, you will be contacted separately about payment when the proofs are issued; please follow the instructions in that e-mail. Arrangements for payment must be made before your article is published. For a complete list of **Publication Fees**, including supplemental material costs, please visit ourwebsite.

Corresponding authors may join or renew ASM membership to obtain discounts on publication fees. Need to upgrade your membership level? Please contact Customer Service at Service@asmusa.org.

Thank you for submitting your paper to Microbiology Spectrum.

Response to Reviewers

Editor comments:

Please note that ASM Journals have an open data policy and requires a 'Data Availability Statement' that provides information where readers (and reviewers) can access data (e.g. RNA-seq, proteomics etc). More information can be found here: https://journals.asm.org/open-data-policy

Reply：Thank you for your comments. The raw data of RNA-seq and DNA-seq were uploaded to NCBI and will be released on November 1st. The raw data of proteomics was uploaded to iProX. Specific information of the data was shown in Data Availability Paragraph after Materials and Methods.

Reviewer comments:

Reviewer #1 (Comments for the Author):

In this manuscript, the authors explore an association between glycine metabolism and novobiocin susceptibility in E. coli. While novobiocin is not currently used as an antimicrobial agent and is not viewed as effective against Gram negative pathogens, drug repurposing efforts merit exploration of orphan drug activity in novel contexts. In this study, the authors use a combination of transcriptomics, proteomics, site-directed mutagenesis and mass-spectrometry to determine that the basis for lack of efficacy of novobiocin against E. coli is related to limited uptake of the drug across the inner-membrane. This finding alone is novel as it is generally thought that the barrier to activity is, in part, through outer-membrane mediated exclusion. Evidence is presented that suggests transport is associated with glycine acquisition, can be modulated by glycine metabolism and is facilitated by the major glycine transporter CycA. While this is a complex story, it is presented in a way that is somewhat easy to follow.

Major comments:

Since this story is quite complex, with numerous relevant intersecting metabolic pathways, regulators and transporters, it would be highly beneficial to include a figure that assimilates all of the key points that the authors are looking to convey.

Reply：Thank you for your comments. Figure 7 was added into the manuscript, which assimilates all of the key points that we are looking to convey.

Tables in the manuscript have repetitive information and can be highly condensed. Tables 3, 6 & 8 should be combined. Table 5 can be eliminated as it does not provide any information that is not found elsewhere in the manuscript.

Reply：Thank you for your comments. Tables 3, 6 & 8 of the original manuscript were combined into Table 3 of the revised MS. Table 5 provided the MICs of the reverse mutants of *E. coli* W3110 △*glyA* to NOV.

The authors have used RNA-seq and proteomics to test and generate key ideas within this manuscript. However, very little of these data are shown. It is recommended that the authors include some representation of these global datasets, such as volcano plots. It is also essential that

the authors perform and present more rigorous analysis of these data, for example through the use of principal component analyses.

Reply：Thank you for your comments. We provided the volcano plots (Figure 2 and 4) and KEGG enrichment scatter plot (Figure 3) to represent those global datasets. PCA maps of RNA-seq and proteomics for principal component analyses were provided with Figure S1 and Figure S2. We performed and present more rigorous analysis of these data in the text.

There is insufficient description of read depth or thresholds for read quality in RNA-seq and full genome sequencing.

Reply：Thank you for your comments. In RNA-seq, read depth is 23-33 M reads and the coverage of the tested samples compared to the reference genome is more than 96%. Differentially expressed genes were selected only if the false discovery rate q-value ≤ 0.05 and fold-change ≥ 2. In full genome sequencing, read depth is 725× with 13-19 M reads. The coverage of the tested samples compared to the reference genome is more than 99%.

For mass spec results, there is no description of extraction controls nor methods and standards for quantification, which are essential for quantitative MS from cellular extracts. Raw data should be provided as supplemental information.

Reply：Thank you for your comments. Standard curve was established, and the methods and standards for quantification were added into the section of Materials and Methods of the revised MS. The raw data of Figure 3 was provided as Table S1.

Figure legends are not sufficiently informative. Please add enough information in the figure legends for readers to understand what the data represent. For Figure 3, provide additional information regarding statistical analysis, "P values were calculated using t tests" is not adequate. There are numerous versions of and approaches for t tests.

Reply：Thank you for your comments. More information was provided into the revised figure legends. For Figure 3, two independent samples t test was used to compare the average NOV contents between the two groups.

Minor points:

Line 69, start new paragraph at "CycA is an inner membrane protein..."

Reply：Thank you for your comments. We started new paragraph at "CycA is an inner membrane protein...".

Line 89, change "reports" to "report"

Reply：Thank you for your comments. We changed "reports" to "report".

Lines 90, 106 and 259, change "minimum" to "minimal"

Reply：Thank you for your comments. We changed "minimum" to "minimal".

Line 100, need a citation for "Since cysB was shown to be related to NOV susceptibility in *E.coli*..."

Reply：Thank you for your comments. A citation was added in the text.

Lines 127, 128, 139 and 146, delete "Further"

Reply：Thank you for your comments. We deleted "Further".

Line 136, provide some rationale for why hslJ was examined

Reply：Thank you for your comments. We provided the rationale for why hslJ was examined (Since the *cysB-regulated* gene *hslJ* was shown to be related to NOV susceptibility in *E. coli* (29), it was worthwhile to examine *hslJ* in the bacteria.).

Line 201, change "level of" to "abundance within the"

Reply：Thank you for your comments. We changed "level of" to "abundance within the".

Line 202, change "assimilation" to "acquisition"

Reply：Thank you for your comments. We changed "assimilation" to "acquisition".

Line 229, change "recovered" to "reversed"

Reply：Thank you for your comments. We changed "recovered" to "reversed".

Line 367, there is incorrect reference to Table 2, there is no table provided that gives the described information

Reply：Thank you for your comments. We deleted the incorrect reference to Table 2 and provided description information here.

Line 379, it is not clear what is meant by "* database"**

Reply：Thank you for your comments. We replaced "*** database" with " Escherichia_coli_uniprot_2020_7_2.fasta(9432 sequences) database".

November 3, 2022

Prof. Jiaoyu Deng
Key Laboratory of Special Pathogens and Biosafety, Wuhan Institute of Virology, Chinese Academy of Sciences
middle street of xiaohongshan, NO.44
Wuhan, Hubei 430071
China

Re: Spectrum02501-22R1 (CycA-dependent glycine assimilation is connected to novobiocin susceptibility in Escherichia coli)

Dear Prof. Jiaoyu Deng:

Your manuscript has been accepted, and I am forwarding it to the ASM Journals Department for publication. You will be notified when your proofs are ready to be viewed.

The ASM Journals program strives for constant improvement in our submission and publication process. Please tell us how we can improve your experience by taking this quick Author Survey.

As an open-access publication, Spectrum receives no financial support from paid subscriptions and depends on authors' prompt payment of publication fees as soon as their articles are accepted. You will be contacted separately about payment when the proofs are issued; please follow the instructions in that e-mail. Arrangements for payment must be made before your article is published. For a complete list of **Publication Fees**, including supplemental material costs, please visit ourwebsite.

ASM policy requires that data be available to the public upon online posting of the article, so please verify all links to sequence records, if present, and make sure that each number retrieves the full record of the data. If a new accession number is not linked or a link is broken, provide production staff with the correct URL for the record. If the accession numbers for new data are not publicly accessible before the expected online posting of the article, publication of your article may be delayed; please contact the ASM production staff immediately with the expected release date.

Corresponding authors may join or renew ASM membership to obtain discounts on publication fees. Need to upgrade your membership level? Please contact Customer Service at Service@asmusa.org.

Thank you for submitting your paper to Spectrum.

Sincerely,

Jeffrey Gralnick
Editor, Microbiology Spectrum

Journals Department
American Society for Microbiology
1752 N St., NW
Washington, DC 20036
E-mail: spectrum@asmusa.org

Supplemental Material: Accept
